# Differential Cytokine Profiles in Prostate Cancer Under Treatment: Implications for Prognosis and Synergistic Therapy Design

**DOI:** 10.3390/cancers18060967

**Published:** 2026-03-17

**Authors:** Aaron E. Katz, Maryann Johnson, Lora J. Kasselman, Saba Ahmed, Ankita Srivastava, David J. Grossfeld, Heather A. Renna, Kathleen Li, Allison B. Reiss

**Affiliations:** 1 Department of Urology, NYU Grossman Long Island School of Medicine, Mineola, NY 11501, USA; aaron.katz@nyulangone.org (A.E.K.); katie_li@urmc.rochester.edu (K.L.); 2 Department of Foundations of Medicine, NYU Grossman Long Island School of Medicine, Mineola, NY 11501, USA; johnson.maryann10@gmail.com (M.J.); saba.ahmed@nyulangone.org (S.A.); ankita.srivastava@nyulangone.org (A.S.); grossfelddavid@gmail.com (D.J.G.); heather.renna@nyulangone.org (H.A.R.); 3 Department of Medical Sciences, Hackensack Meridian School of Medicine, Nutley, NJ 07110, USA; lora.kasselman@hmhn.org; 4 Department of Medicine, NYU Grossman Long Island School of Medicine, Mineola, NY 11501, USA

**Keywords:** prostate cancer, cytokine, interleukin, cryotherapy, inflammation, prostatectomy

## Abstract

**Background/Objectives**: Localized prostate cancer may be treated with total cryotherapy, focal cryotherapy, stereotactic body radiotherapy (SBRT), or radical prostatectomy (RP). However, the immune response to these therapies is not fully understood despite its potential importance in determining extent and timing of recovery, disease control and cancer recurrence rate. This exploratory study measured cytokine expression changes in the urine and blood of prostate cancer patients as a means of monitoring immune response to these four alternative treatments. **Methods**: Urine and blood multiplex ELISA cytokine assays were performed in 37 men with histologically confirmed prostate adenocarcinoma before, 2 weeks after, and 3 months after therapy. **Results**: Treatment method alone significantly affected levels of plasma but not urine cytokines. Both plasma and urine showed significant changes across visit number and significant interactions between treatment and visit number for some cytokines. In plasma, SBRT was associated with the highest cytokine levels when compared to RP and cryotherapy. Urinary IL-8 levels increased over time following cryoablation, whereas they remained relatively stable across visits after SBRT and RP (β = 1.51, 95% CI [0.89–2.13], *p* < 0.001). Urinary IL-6 levels reached their highest point at visit three following both SBRT and cryoablation, whereas after RP, the peak occurred earlier at visit two (β = 0.07, 95%CI [0.04–0.11], *p* < 0.001). The pattern of plasma levels of IL-10 differed by time elapsed after treatment depending upon treatment group (β = −0.05, 95%CI [−0.08–−0.01], *p* = 0.005). **Conclusions:** These findings show that the prostate cancer treatment method employed may affect post-operative inflammatory mechanisms. This small study encourages expansion to determine the prognostic utility of urine and plasma cytokine expression patterns based on prostate cancer treatment and time elapsed following treatment. Understanding these changes may inform a personalized medicine approach to prostate cancer immunotherapies.

## Simple Summary

Localized prostate cancer can be managed using total or focal cryotherapy, stereotactic body radiotherapy (SBRT), or radical prostatectomy (RP). The immune response to these therapies remains poorly understood despite its role in recovery, disease control, and recurrence. This exploratory study found distinct urine and plasma cytokine patterns by treatment and visit, highlighting treatment-specific inflammatory responses and their potential prognostic value. We documented sustained urinary IL-8/IL-10 rises post-cryoablation, and delayed IL-6 peaks with SBRT/cryoablation vs. earlier RP peaks. Our findings underscore key targets for larger future studies.

## 1. Introduction

There are multiple treatment options for localized prostate cancer, including radical prostatectomy (RP), partial cryoablation (PC), total cryoablation (TC), and conventional or stereotactic body radiotherapy (SBRT) [1,2,3]. In contrast to surgical removal of the entire prostate via RP, cryotherapy and SBRT may reduce the risk of damage to surrounding tissue, since cryotherapy is a minimally invasive procedure using the controlled application of extreme cold and SBRT uses a robotic system for targeted delivery of high doses of radiation [4]. However, the systemic inflammatory response to each of these therapies is not yet well characterized. Currently available laboratory-based techniques to obtain blood and body fluid cytokine profiles present a means of assessing the inflammatory state non-invasively and can be applied in prostate cancer and many other disease states [5,6,7]. The pattern of cytokine release may affect wound healing and post-operative recovery, but there is mixed data on the association between inflammatory disease and the biochemical recurrence of prostate cancer [8,9]. Furthermore, several cytokines have been identified as having both pro- and anti-tumor effects, further complicating discussion of the clinical implications of post-procedure cytokine outcomes.

Cytokines can be broadly grouped into three categories: inflammatory, anti-inflammatory, and dual function (where cytokines can be inflammatory or anti-inflammatory). Inflammatory cytokines include tumor necrosis factor (TNF)-α, interferons (IFN) such as IFN-γ, and interleukins (IL)-1, IL-4, IL-5, IL-8, IL-9, IL-14 and IL-15. Anti-inflammatory interleukins include IL-7, IL-10, IL-30 and IL-37 [10,11]. IL-6, IL-13, and IL-17 can exhibit both inflammatory and anti-inflammatory properties [12,13,14].

Since the immune environment is key to biochemical recurrence-free survival, further studies are needed to explore the influence of treatment choice on the cytokines that help to shape this environment [15]. This information may inform future prostate cancer treatment paradigms about the combination of surgical or procedural treatment with immunotherapy.

The primary aim of this study was to identify candidate cytokines demonstrating treatment-specific and time-dependent expression patterns following therapy for localized prostate cancer. Secondary aims were to determine whether urinary cytokine patterns would substantially differ from plasma cytokine patterns because these differences may reflect that urinary cytokines pick up on localized prostatic inflammation distinct from systemic plasma cytokine changes that more likely indicate systemic immune activation and broader inflammatory signaling. This rationale underpins our dual compartmental sampling approach. Compared to plasma, urine cytokines remain underexplored and identification of crucial cytokines that may serve as noninvasive recurrence predictors can direct prioritization in future prognostic and recurrence-focused investigations [16].

Although individual therapies have been shown to modulate inflammatory cytokines, comparative data examining their temporal immune signatures across treatment modalities and biological compartments remain limited, particularly with the increasing use of focal therapies such as cryoablation and stereotactic body radiation therapy (SBRT) [17]. Plasma cytokine changes primarily reflect systemic immune responses and whole-body inflammatory signaling, including acute phase mediators such as IL-1, IL-6, and TNF-α, which can manifest clinically as fever, fatigue, and malaise [18]. In some contexts, these cytokines may also contribute to tumor progression or influence recurrence risk, while radiation therapy may induce broader immune activation [19]. In contrast, urinary cytokines may better reflect localized prostate or pelvic tissue inflammation and injury [20,21].

A clearer understanding of treatment-specific cytokine trajectories may therefore provide insight into wound healing, tumor microenvironment remodeling, and the risk of biochemical recurrence [22]. This exploratory study, hypothesis-generating in nature, characterized and compared longitudinal changes in urine and plasma cytokine profiles in patients undergoing total cryotherapy, partial cryotherapy, SBRT, or radical prostatectomy.

## 2. Materials and Methods

### 2.1. Study Design and Subjects

This study was conducted in accordance with the principles of the Declaration of Helsinki and was approved by the Winthrop University Hospital Institutional Review Board (i18-01696-2). Informed consent was obtained from all individual participants included in the study.

This was a phase II, non-sham, non-randomized, exploratory four-armed study that enrolled 37 patients diagnosed with histologically confirmed prostate adenocarcinoma and undergoing one of the following treatments: TC (*n* = 10), PC (*n* = 9), SBRT (*n* = 8), and RP (*n* = 10). Patients were recruited at time of consultation. They were then asked to provide urine and blood samples at the initial screening visit, 2 (±1) weeks post-treatment, and 3 months post-treatment for a total participation of up to 4 months. Patients with evidence of metastasis, previous prostate cancer treatment, substance or alcohol use disorder that would impair participation, or known immunologic diseases were excluded. This exploratory study intentionally included all eligible patients (*n* = 37 across treatments) rather than a homogeneous subgroup (e.g., *n* = 8 per modality) to maximize detection of candidate cytokines with differential patterns, powering future targeted validation studies.

Patient allocation was not influenced in any way by study participation. Prostate cancer treatment allocation at the Winthrop University Hospital is a personalized process guided by multidisciplinary teams (including urologists, radiation oncologists, and medical oncologists) that evaluate the cancer’s severity (based on tumor stage), the patient’s overall health, and personal preferences. Treatment decisions are based on risk stratification (low, intermediate, high, or metastatic), which informs whether the goal is curative (such as surgery, radiation, or cryotherapy) or focused on disease management (such as active surveillance) [23].

### 2.2. Blood and Urine Collection

Whole blood was collected into lavender-topped tubes containing ethylenediaminetetraacetic acid (EDTA) as an anticoagulant and centrifuged at 1000–2000× *g* for 10 min in a refrigerated centrifuge (Beckmann Coulter (4 °C)). The resulting plasma, separated as supernatant, was promptly transferred into clean polypropylene tubes using a Pasteur pipette (MilliporeSigma, St. Louis, MO, USA). Samples were kept at 2–8 °C during handling and divided into 0.5 mL aliquots for storage at −20 °C until the time of analysis.

Urine samples were self-voided and collected in 10 mL collection tubes (MilliporeSigma) using the midstream clean-catch procedure. Samples were placed on ice immediately and transferred to the laboratory for preparation. Upon arrival, samples were centrifuged at 1500–3000× *g* for 10–15 min at 4 °C to remove cellular debris and particulates. The supernatant was aliquoted into sterile polypropylene cryovials (Thermofisher, Waltham, MA, USA) and stored at −80 °C until further analysis. They were used for the ELISA experiments on the same day of collection whenever possible, or after a single freeze-thaw cycle. Frozen samples were thawed once and gently mixed prior to analysis, ensuring sample stability and minimizing cytokine degradation.

### 2.3. Human Cytokine Panel

The Human Inflammatory Cytokines Multi-Analyte ELISArray Kit MEH-004A ((Qiagen, Germantown, MD, USA), which analyzes a panel of 12 pro-inflammatory cytokines simultaneously under uniform conditions, was used to analyze all specimens following the manufacturer’s instructions. Each ELISArray microplate carried biological samples, in addition to positive and negative control samples. Standards and reagents were freshly prepared before each experiment. The cytokines detected by this array are IL-1α, IL-1β, IL-2, IL-4, IL-6, IL-8, IL-10, IL-12, IL-17A, IFN-γ, TNF-α, and granulocyte-macrophage colony-stimulating factor (GM-CSF).

### 2.4. Statistical Analysis

Data were tested for normality using Shapiro–Wilk tests. Statistical analysis of data was performed using mixed effects regression models for each cytokine in both urine and plasma. Each cytokine was regressed separately on treatment and visit, along with an interaction term for treatment and visit. SBRT treatment and visit one were set as references for each of treatment and visit, respectively. A random effect term for participants was included in each model. Notably, no covariates were included in these models as we were focusing on identifying candidate cytokines only. Model assumptions were tested using visualizations of residuals. Model results were reported as the estimate and 95% confidence interval. Significant overall effects were followed with analyses of all contrasts using estimated marginal means (least squares means). Significance level was set at the 0.05 level, with trends identified between 0.05 and 0.10, with no adjustment for multiple hypothesis-testing since this was an exploratory study. Due to the exploratory nature of the study, all results are interpreted with caution and will be used for identifying promising cytokines and powering future studies. All visualizations and analyses were conducted using R statistical software version 4.5.1 from R Foundation for Statistical Computing, Vienna, Austria at https://www.R-project.org/ (accessed on 10 October 2025).

## 3. Results

### 3.1. Demographics

Demographic characteristics are reported in Table 1.

### 3.2. Urine and Plasma Cytokines

**Figure 1:**
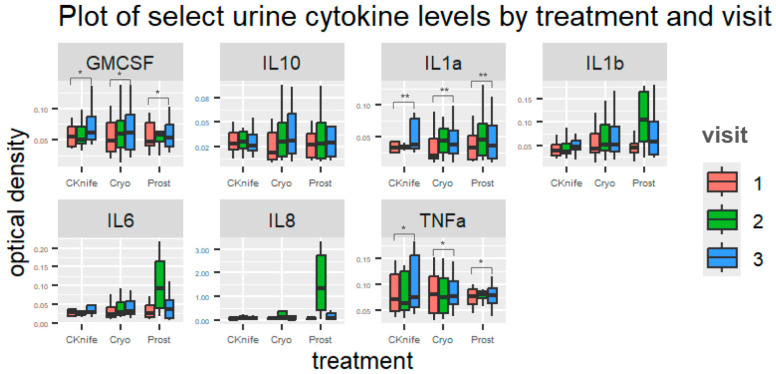
Plot of urine cytokine levels by cytokine, treatment type and visit number. Data visualized using boxplots; y-axis represents cytokine optical density levels. Red boxes represent visit one, green boxes represent visit two, and blue boxes represent visit three. CKnife = cyber knife surgery; Cryo = cryoablation surgery; Prost = radical prostatectomy. * *p* < 0.05 and ** *p* < 0.01.

**Figure 2:**
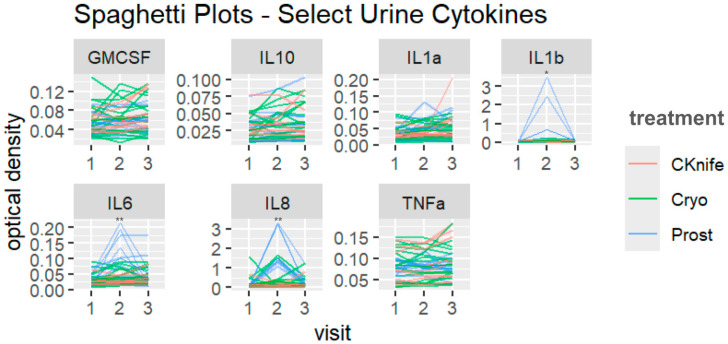
Plot of urine cytokine levels by cytokine, treatment type and visit number. Both treatment and time are represented in all graphs. Data visualized using spaghetti plots; y-axis represents cytokine optical density levels. Red lines represent visit one, green lines represent visit two, and blue lines represent visit three. CKnife = cyber knife surgery; Cryo = cryoablation surgery; Prost = radical prostatectomy. * *p* < 0.05 and ** *p* < 0.01.

**Figure 3:**
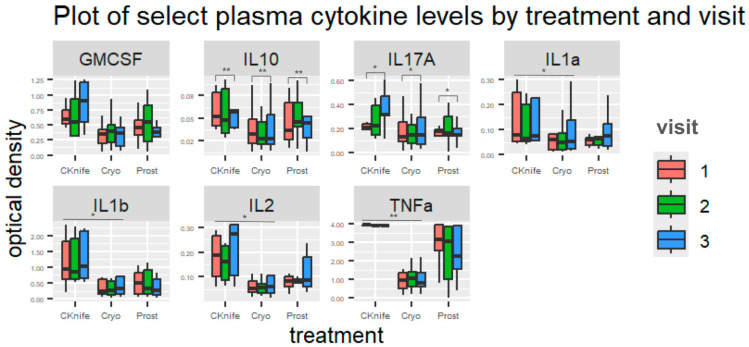
Plot of plasma cytokine levels by cytokine, treatment type and visit number. Data visualized using boxplots; y-axis represents cytokine optical density levels. Red boxes represent visit one, green boxes represent visit two, and blue boxes represent visit three. CKnife = cyber knife surgery; Cryo = cryoablation surgery; Prost = radical prostatectomy. * *p* < 0.05 and ** *p* < 0.01.

**Figure 4:**
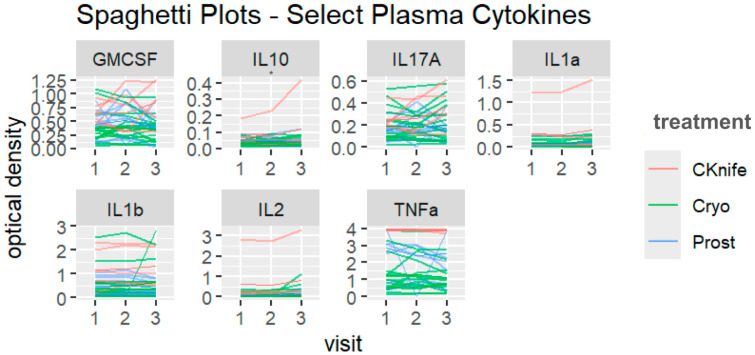
Plot of plasma cytokine levels by cytokine, treatment type and visit number. Both treatment and time are represented in all graphs. Data visualized using spaghetti plots; y-axis represents cytokine optical density levels. Red lines represent visit one, green lines represent visit two, and blue lines represent visit three. CKnife = cyber knife surgery; Cryo = cryoablation surgery; Prost = radical prostatectomy. * *p* < 0.05.

Both treatment and/or visit (time after treatment) were significantly associated with changes in some urine (Figure 1 and Figure 2) and plasma (Figure 3 and Figure 4) cytokine levels. Additionally, there were some significant interactions between treatment and visit in some cytokines. Nonsignificant tests were not recorded here.

### 3.3. Cytokine Levels Associated with Treatment Type

There were no significant differences in *urine* cytokine levels among any treatment conditions.

Overall, SBRT was associated with the highest levels of *plasma* cytokines when compared to RP and cryotherapy (SBRT set as reference group). With respect to specific cytokines, plasma GM-CSF, IL-1α, IL-1β, IL-2, and TNF-α displayed differences across treatment types: GM-CSF (β = −0.26, 95%CI [−0.53–0.01], *p* = 0.062 for SBRT vs. cryotherapy); **IL-1α**, (β = −0.22, 95%CI [−0.43–−0.01], *p* = 0.038 for SBRT vs. cryotherapy and β = −0.22, 95%CI [−0.43–−0.01], *p* = 0.062 for SBRT vs. RP); **IL-1β** (β = −0.67, 95%CI [−1.29–−0.04], *p* = 0.036 for SBRT vs. cryotherapy and β = −0.22, 95%CI [−1.36–0.03], *p* = 0.059 for SBRT vs. RP); **IL-2** (β = −0.51, 95%CI [−0.98–−0.04], *p* = 0.034 for SBRT vs. cryotherapy and β = −0.51, 95%CI [−1.03–0.01], *p* = 0.056 for SBRT vs. RP); and **TNF-α** (β = −2.68, 95%CI [−3.70–−1.66], *p* < 0.001 for SBRT vs. cryotherapy and β = −1.07, 95%CI [−2.20–0.06], *p* = 0.063 for SBRT vs. RP).

### 3.4. Cytokine Levels Associated with Visit Number

Overall, visit 3 was associated with the highest levels of *urine* cytokines when compared to visit two and visit one (visit one set as reference group). Urine GM-CSF, IL-1α, and TNF-α displayed differences across visit numbers: **GM-CSF** (β = 0.02, 95%CI [0.00–0.03], *p* = 0.022 for visit three vs. visit one); **IL-1α** (β = 0.03, 95%CI [0.01–0.05], *p* = 0.009 for visit three vs. visit one); **TNF-α** (β = 0.02, 95%CI [0.00–0.03], *p* = 0.035 for visit three vs. visit one).

Overall, visit three was associated with the highest levels of *plasma* cytokines when compared to visit two and visit one (visit 1 set as reference group). Plasma IL-10, IL-17a, and IL-1α displayed differences across visit numbers: **IL-10** (β = 0.05, 95%CI [0.02–0.08], *p* = 0.001 for visit three vs. visit one); **IL-17α** (β = 0.1, 95%CI [0.02–0.19], *p* = 0.022 for visit three vs. visit one); and IL-1α (β = 0.04, 95%CI [−0.00–0.09], *p* = 0.075 for visit three vs. visit one).

### 3.5. Cytokine Levels Associated with Treatment Type and Visit Number

Overall, *urine* IL-10, IL-1β, IL-6, and IL-8 displayed significant trends towards interactions between treatment and visit. For IL-10, cryoablation cytokine levels increased across visit numbers but remained relatively flat for SBRT and RP groups (β = 0.01, 95%CI [−0.00–0.02], *p* = 0.079). For **IL-1β**, SBRT cytokine levels dipped at visit two but increased at visit two for cryoablation and RP groups (β = 0.63, 95%CI [0.14–1.11], *p* = 0.012). For **IL-6**, SBRT and cryoablation cytokine levels peaked at visit three but for RP they peaked at visit two (β = 0.07, 95%CI [0.04–0.11], *p* < 0.001). For **IL-8**, cryoablation cytokine levels increase across visits but remain relatively flat across visits for SBRT and RP (β = 1.51, 95%CI [0.89–2.13], *p* < 0.001).

Overall, *plasma* GM-CSF, IL-10, and IL-17α displayed significant trends towards interactions between treatment and visit. For GM-CSF, cryoablation cytokine levels were lowest at visit three, RP cytokine levels peaked at visit two, and SBRT cytokine levels peaked at visit three (β = −0.24, 95%CI [−0.53–0.04], *p* = 0.096). For **IL-10**, cryoablation and SBRT cytokine levels peaked at visit three but RP cytokine levels peaked at visit two (β = −0.05, 95%CI [−0.08–−0.01], *p* = 0.005). For IL-17α, SBRT and cryoablation cytokine levels peaked at visit three but for RP they peaked at visit two (β = −0.09, 95%CI [−0.19–0.01], *p* < 0.065).

## 4. Discussion

Understanding changes in the inflammatory response to focal prostate cancer treatments and throughout the healing process may be important to create personalized therapeutic approaches and improve patient outcomes [24,25,26,27].

Our choice of cytokines was selected based upon the cytokines measured clinically by urologists in prostate carcinoma patients to distinguish between local vs. advanced disease and to characterize cachexia and systemic inflammation [18,28,29,30]. Additionally, the final panel was determined by the availability of analytes within a commercially available pre-defined multiplex cytokine assay, which placed some practical constraints on our selection. Previous research has investigated post-procedure changes in several cytokines. For example, RP has been shown to increase the serum level of inflammatory cytokines such as IL-8, IL-6, IL-10, IFN-γ and IL-12P70 while lowering the level of other inflammatory cytokines IL-4, IFN-α, TNF-α and IL-1β [31]. Cryosurgery generates a cryo-immune anti-tumor response with an increase in IFN-γ and TNF-α [32,33]. However, cryosurgery can also increase circulating transforming growth factor (TGF)-β, which may promote tumor recurrence [34,35]. Finally, SBRT can increase IFN-γ and elicit a strong immune response [1,36].

These results contribute to the mixed findings of clinical studies on immune responses to prostate cancer treatment in humans. For example, cryosurgery can have both immunostimulatory and immunosuppressive effects, depending on the study. When an immunostimulatory effect was observed, these responses were short-lived. In a small group of 20 patients with high-risk prostate cancer undergoing cryoablation, serum levels of TNF-α and IFN-γ were significantly higher four weeks post-treatment, with no changes in IL-4 or IL-10 levels [31]. The Th1/Th2 ratios, estimated by the IFN-γ/IL-4 ratio, increased four weeks after treatment, but decreased by eight weeks post-cryoablation. Cytolytic activity against the LNCaP androgen-sensitive human prostate adenocarcinoma cell line was noticeable four weeks post-treatment but had diminished by eight weeks. In a study of twelve patients undergoing prostate cryosurgery, circulating CD4+, CD25+, CD127− T cells, or regulatory T cells with a suppressive effect on the immune system were evaluated before and one-month post-treatment [37]. These regulatory cells significantly decreased four weeks after cryosurgery, and this effect persisted up to eight weeks after treatment. However, the suppressive activity of these T-cells increased in eight of the twelve patients, and cancer recurred in two patients six months post-treatment.

Our findings, though exploratory in nature, indicate differing patterns of urine and plasma cytokine levels across treatment groups and visit number. These results suggest that cytokine responses depend upon the type of procedure and time, as well as highlight potential foci for future powered studies. We may theorize that the rise in IL-10 with cryoablation is associated with a shift toward wound healing [38]. Urine IL-1β, IL-6 and IL-8 saw higher levels within the cryoablation group at different timepoints compared to SBRT and RP. Sustained elevations (IL-10, IL-8) suggest the possibility of prolonged tissue ablation effects [39].

Plasma IL-10 was higher at visit three in the cryoablation and SBRT groups compared to RP. Notably, IL-1, IL-6, and IL-8 are pro-inflammatory cytokines functionally linked to prostate cancer progression, high PSA and the transition to androgen-insensitive castrate resistance [40]. The IL-1 family has also been identified as potential biomarkers for prostate cancer stage and prognosis, with IL-1α implicated in tumor angiogenesis, androgen insensitivity, and metastasis [41]. Moreover, several treatment-specific differences in cytokine levels were detected in plasma only. Previous research has identified urine cytokines as potential indicators of renal inflammation and clear cell renal carcinoma, but not for systemic inflammation in a population of women with low levels of inflammation [42,43,44]. Prostate cells may release cytokines into prostatic fluid and ultimately into urine, and therefore the isolated elevation of urinary cytokines may indicate localized inflammation [45]. Urine cytokines are therefore clinically relevant as they can act as non-invasive biomarkers indicative of regional tumor inflammation, immune responses, and disease progression, complementing serum measurements and perhaps improving sensitivity by detecting microenvironment signals that serum alone may dilute [40,46,47]. Cytokines rising early may be a result of tissue injury and immune activation in contrast to later cytokine peaks that may reflect resolution and healing. While timing of cytokine changes might distinguish an initial inflammatory phase from a subsequent immunoregulatory phase, further studies incorporating cellular immune profiling would be required to make these distinctions [48].

However, more research is needed to examine whether these urine cytokines indicate prostatic or renal pathologies. Chakravarty et al. found that greater pelvic inflammation could promote the growth of pre-existing prostate cancer [9]. Further, more research is needed to determine whether local cytokine elevations insufficient to be detected in plasma are significantly associated with surgical outcomes and prostate cancer recurrence. Overall, higher radiation doses to the pelvic area are associated with a greater inflammatory cytokine response [49]. Limited previous research has shown independence in serum and urine cytokine levels after prostate cancer intensity-modulated radiotherapy, but it is not known whether this is representative or associated with recurrence [50]. Future studies could also illuminate how physical dose delivery translates into biological immune signaling during radiation treatment. The importance of radiobiological research to link dosimetry with biological response is increasingly recognized [51].

Limitations of this study include a relatively small sample size of 37 patients who were not randomly assigned to treatment groups. As an exploratory study, all trends and significant results remain preliminary and must be replicated using future fully powered studies, though this study provided evidence identifying which cytokines to focus on in future studies. Patients were not matched based on characteristics, such as race, age, and disease stage/grade. Patients were also not matched by initial cytokine levels; these differences in starting cytokine level may contribute to the observed significant differences by treatment type. Urinary cytokine levels were not normalized to creatinine in this study, as our primary focus was on absolute concentration changes over time. However, we recognize that creatinine normalization would strengthen longitudinal comparisons by accounting for hydration variability and urine dilution. Focal and total cryotherapy patients were grouped together, so it is not known whether a focal approach may affect post-operative cytokine levels. Focal and total cryotherapy may elicit distinct biological impacts, particularly in terms of immune activation and systemic cytokine release. However, given the limited sample sizes per subgroup, we pooled these cryotherapy arms for primary analyses to enhance statistical power. In this exploratory study, detailed radiotherapy parameters, including total radiation dose, fractionation schedule, and biologically effective dose (BED), were not systematically collected and are therefore not reported. This was a small observational study and, unfortunately, we do not have complete demographic data; nonetheless, we recognize that in the future it will be important to analyze any relationships between sociodemographic characteristics and cytokine levels following treatment.

## 5. Conclusions

In this study, changes in cytokine profile in prostate cancer patients undergoing common treatment modalities were compared among treatment groups and changes over time were assessed. Given the limited sample size and broad profiling without a pre-defined target, the present work should be considered hypothesis-generating, intended to inform future confirmatory studies with larger, independent cohorts.

The results, while exploratory, demonstrated differing patterns of urine and plasma cytokine levels across treatment groups and visit number, indicating the need for further confirmation and future elaboration on why different cytokines and locations (plasma versus urine) respond differently to different treatment types. Additional and expanded investigations are needed to determine whether these urinary cytokine changes are associated with prostate cancer recurrence, and whether focal approaches significantly affect changes in urine and plasma cytokine levels when compared to the total or radical approach. Ultimately, this exploratory study may provide a foundation for hypothesis-driven studies aimed at advancing a personalized medicine approach to prostate cancer care and treatment selection.

## Figures and Tables

**Table 1:** Demographic characteristics of the sample.

	Cknife (*n* = 11)	Cryo (*n* = 32)	Prost (*n* = 10)	Overall (*n* = 53)
Age at Treatment (years)				
Mean (SD)	69.1 (5.79)	68.8 (7.44)	60.8 (6.47)	67.3 (7.54)
Median [Min, Max]	67.9 [61.2, 78.9]	69.3 [52.1, 80.2]	62.4 [50.2, 68.81]	67.8 [50.2, 80.2]
Race				
Black	0 (0%)	1 (3.1%)	3 (30.0%)	4 (7.5%)
Hispanic	0 (0%)	0 (0%)	1 (10.1%)	1 (1.9%)
White	3 (27.3%)	29 (90.6%)	6 (60.0%)	38 (71.7%)
White/Hispanic	0 (0%)	1 (3.1%)	0 (0%)	1 (1.9%)
Unknown	8 (72.7%)	1 (3.1%)	0 (0%)	9 (17.0%)
Pre-trial PSA levels (ng/mL)				
Mean (SD)	NA (NA)	6.32 (3.66)	6.94 (3.93)	6.55 (3.69)
Median [Min, Max]	NA [NA, NA]	5.31 [1.03, 14.7]	5.00 [4.00, 13.9]	5.16 [1.03, 14.7]
Missing	11 (100%)	17 (53.1%)	1 (10.0%)	29 (54.7%)

CKnife = cyber knife surgery; Cryo = cryoablation surgery; Prost = radical prostatectomy.

## Data Availability

The full dataset is available from the corresponding author, upon motivated request.
